# Neuropeptide Y Stimulates Proliferation and Migration of Vascular Smooth Muscle Cells from Pregnancy Hypertensive Rats via Y1 and Y5 Receptors

**DOI:** 10.1371/journal.pone.0131124

**Published:** 2015-07-01

**Authors:** Ping Zhang, Ying-Xin Qi, Qing-Ping Yao, Xiao-Hu Chen, Guo-Liang Wang, Bao-Rong Shen, Yue Han, Li-Zhi Gao, Zong-Lai Jiang

**Affiliations:** Institute of Mechanobiology and Biomedical Engineering, School of Life Sciences and Biotechnology, Shanghai Jiao Tong University, Shanghai, China; UC San Diego, UNITED STATES

## Abstract

The increased proliferation and migration of vascular smooth muscle cells (VSMCs) play important roles in pathophysiological remodeling of arteries during hypertension in pregnancy. However, the mechanisms involved in this process remain unclear. We hypothesized that Neuropeptide Y (NPY), which is a potent mitogenic peptide, participates in modulating proliferation and migration of VSMCs during hypertension in pregnancy. Using pregnant hypertensive rats, induced by intraperitoneal injection of L-nitro-arginine methylester (L-NAME), the plasma concentration of NPY was detected. Open angle, which reflects the non-uniform remodeling with high sensitivity, was used to detect the pathophysiological vascular remodeling *in vivo*. The results revealed that NPY concentration and artery open angle were both significantly increased in rats with hypertension in pregnant. The underlying mechanism of elevated NPY on vascular remodeling were further analyzed by using cultured VSMCs *in vitro*. In cultured VSMCs, NPY most effectively stimulated the migration and proliferation of VSMCs at 10^-6^ mol/L, similar to the plasma concentration in L-NAME hypertension in pregnant rats. NPY up-regulated the expressions of both Y1 and Y5 receptors, increased the phosphorylations of STAT3 on Tyr705 and Ser727 residues, and induced the expression of c-Fos. The NPY-induced VSMCs proliferation was reduced by Y5 receptor antagonist, and fully blocked by combinations with other antagonist, such as Y2+Y5, Y1+Y5, and Y1+Y2+Y5. In contrast, the NPY-induced VSMC migration was blocked by either Y receptor antagonist or any combination of Y receptor antagonists. These results suggest that the elevated plasma concentration of NPY during hypertension in pregnancy may induce VSMC proliferation mainly via Y5 receptor, which subsequently modulate STAT3 and c-Fos signaling pathways to result in the vascular remodeling. These results also suggest that NPY mainly acts on VSMCs *in vitro* via Y1, Y5 receptors and in vascular tissues *in vivo* via Y5 receptor.

## Introduction

Neuropeptide Y (NPY) is a 36-amino acid polypeptide [1], which highly expresses in the brain, adrenal medulla [2], sympathetic nerves [3], and non-neuronal endothelial cells (ECs) [4]. It has been reported that NPY, as a potent peripheral regulatory peptide, is participated in immune responses, stimulates hyperlipidemia, induces vasoconstriction, as well as regulates the proliferation of various cell types including ECs and vascular smooth muscle cells (VSMCs) through its corresponding receptors [5, 6]. NPY stimulates a class of G-protein-coupled receptors named as Y receptors [7], including six subtypes, i.e. Y1, Y2, Y3, Y4, Y5 and Y6 [6, 7]. The Y1 receptor post-synaptically mediates vasoconstriction and increases the blood pressure, by potentiating norepinephrine induced contraction and VSMC proliferation [5, 8]. Zukowska et al have shown that NPY stimulates Y1 and Y2 receptors and involved in multiple steps of atherogenesis, including EC attachment, migration, proliferation, and differentiation [4]. The Y2 receptor acts alone or works together with the Y5 receptor to potentiate angiogenesis, stimulate proliferation, migration, and capillary tube formation of ECs [9]. All these researches revealed that NPY and its receptors, including Y1, Y2 and Y5, paly important roles in functional regulation of cardiovascular system.

Hypertension in pregnancy, defined as the new-onset hypertension during pregnancy [10], can induce pathophysiological vascular remodeling, which is associated with maternal multisystem involvement, preterm delivery, fetal morbidity and future cardiovascular and metabolic diseases [10, 11]. Researches revealed that the vascular remodeling during hypertension in pregnancy is characterized by the abnormal hypertrophy, proliferation and migration of VSMCs [12]. Studies had shown that various signaling pathways are involved in VSMC dysfunction induced by hypertension in pregnancy, such as Ca^2+^, mitogen-activated protein kinases (MAPKs) [13] and G-protein [14]. However, the molecular mechanism of hypertension in pregnancy induced VSMC proliferation and migration remains to be further elucidated.

It has been proved that neuropeptide, especially the peripheral NPY concentration is increased during hypertension in pregnancy [15]. Furthermore, the plasma concentration of NPY has been proved to be a risk factor in cardiovascular system, and increases in various conditions, such as stress [16], hypertension [17, 18], and congestive heart failure [19]. However, its molecular mechanism has not been fully elucidated. In stromal vascular cells and brain, NPY has been proved to modulate phosphorylation of STAT3 and expression of c-fos [20, 21], which are important signaling molecules also involved in VSMC functions [22]. Since studies have revealed that plasma concentration of NPY is increased during hypertension in pregnancy and NPY is involved in the regulation of VSMC functions, we therefore hypothesized that NPY plays important roles in vascular remodeling during hypertension in pregnancy, which may involve STAT3 and c-fos pathways.

In the present study, L-nitro-arginine methylester (L-NAME), which blocks the nitric oxide synthase, were intraperitoneally injected to pregnant rats to induce hypertension in pregnancy [23, 24]. Then the concentration of NPY, the expression of Y receptors and the thoracic aorta remodeling were examined in vivo. To elucidate the underlying molecular mechanism, we further detected the in vitro effect of NPY on VSMC proliferation and migration, the expression of Y receptors, and analyzed the potential signaling molecules involved in this process. The results may shed some new lights on the roles of NPY in vascular remodeling occurring in hypertension in pregnancy.

## Materials and Methods

All experimental protocols and the investigation were approved by the Animal Research Committee of Shanghai Jiao Tong University, and the Animal care conforms to the Animal Management Rules of China (Documentation 55, 2001, Ministry of Health, China).

### 2.1 Established model of hypertension in pregnancy

Female Sprague—Dawley (SD) rats, weighing 250 ± 10 g, were caged overnight with mature males during oestrum. Next day, vaginal smears were checked for the presence of spermatozoa in the early morning. The time with the presence of spermatozoa in vaginal smear was defined day 0.5 (P0.5) of pregnancy. On day 14.5 (P14.5) of pregnancy, the rats were randomly assigned to two groups. One group was intraperitoneally injected with 0.2 ml L-NAME (27.5 mg, Sigma) every day; the other group was injected with 0.2 ml saline as control. On day 20.5 (P20.5), all rats were executed for the subsequent experiments.

### 2.2 Measurement of blood pressure

The blood pressure was recorded on P12.5 and P20.5 using the tail-cuff method [25] by a programmable sphygmomanometer (BP-98A, Softron). The systolic blood pressure, diastolic blood pressure and mean arterial pressure were recorded.

### 2.3 Radioimmunoassay (RIA) of NPY plasma concentration

On P20.5 of pregnancy, rats were euthanized with sodium pentobarbital at 120 mg/kg. After the animal was sacrificed, the plasma of the two group rats were collected. The NPY concentrations were measuring using NPY RIA kit (HY-098) by Beijing DORUN International Technology Co., Ltd.

### 2.4 Opening angle

Opening angle, which reveals the non-uniform remodeling or anisotropic growth across the arterial wall during hypertension [26, 27], was used to detect the pathophysiological vascular remodeling. On P20.5 of pregnancy, the pregnant rats were sacrificed with sodium pentobarbital at 40 mg/kg. The thoracic aorta was surgically removed, carefully dissected and placed into a Ca^2+^-free Krebs solution (117.9 NaCl, 4.7 KCl, 1.2 KH_2_PO_4_, 25 NaHCO_3_, 1.2 MgSO_4_, 2.5 CaCl_2_, and 11 glucose, composition in mM, pH 7.4). The descendent aorta of 5 mm was cut into 4 equal length rings, and then cut to open rings. The bathing solution was gassed with 5% CO_2_ at 37°C for 30 minutes and photographed by microscope (Olympus SZX16) in the no-load state. The opening angle, defined as the angle between the two lines from the middle point the inner vascular wall to the tips of the inner wall, was analyzed with Image Pro Plus 6.0. The opening angles of the sectors from a vessel were averaged to represent the value for the vessel [27].

### 2.5 VSMCs culture

Primary rat VSMCs were isolated from the thoracic aortas of female SD rats (200 ± 20 g), using an explant method [28]. VSMCs were characterized by immunohistochemial staining for smooth muscle-specific α-actin (DAKO). Cells with more than 95% purity were passaged every 3 to 4 days after trypsinization and passages 4–7 were used in all experiments.

VSMCs were cultured in 24-well plate in DMEM supplemented with 10% FBS and 5% CO_2_ at 37°C. Cells were grown in medium without FBS for 24 hours before confluence. VSMCs were stimulated with different concentrations of recombinant NPY (Human and rat; Abcam) for 24 hours or 48 hours, with ranges across 10^−6^, 10^−8^, 10^−10^ and 10^−12^ mol/L.

Since three kinds of NPY receptors, i.e. Y1, Y2 and Y5, are reported to mediate the functions of cardiovascular system [6, 8], the specific antagonists were used to treat VSMCs for 30 minutes before NPY stimulation in order to analyze the receptors involved in: Y1 receptor antagonist, BIBO 3304 trifluoroacetate (10^−7^ mol/L, SantaCruz); Y2 receptor antagonist, SF 11 (10^−7^ mol/L, SantaCruz); and Y5 receptor antagonist, NPY 5RA972 (10^−7^ mol/L, Tocris). The proliferation, migration and expressions of potential signaling molecules in VSMCs were then detected.

### 2.6 Western Blotting

Thoracic aorta was grinded using homogenizer. The aortic homogenation and cultured VSMCs were lysed with 0.25 M Tris (pH 6.8), 1.5% SDS, and 1% mercaptoethanol on ice. The lysis samples were subjected to electrophoretic separation with 10% SDS polyacrylamide gel electrophoresis and transferred onto NC membrane (Millipore, Schwalbach, Germany) as described earlier [29]. Membranes were pre-incubated with 5% dry-milk powder incubated 1 hour at room temperature and then incubated overnight at 4°C with rabbit polyclonal antibody Y1R (1:500, Abcam), Y5R (1:500, Abcam), STAT3 (1:500, Cell Signaling), P-STAT3 (705) (1:500, Cell Signaling), P-STAT3(727) (1:500, Cell Signaling), c-Fos (1:500, PTG), goat polyclonal antibody NPY2R (1:500, Sigma), rabbit polyclonel antibody PCNA(1:1000, PTG), mouse polyclonal antibody GAPDH (1:1000, PTG), respectively. After incubation with alkaline phosphatase-conjugated secondary antibodies (Jackson Immunoresearch), the signals were visualized by nitroblue tetrazolium—bromochloroindolyl phosphate (Bio Basic, Inc.) and quantified with Quantity One software (Bio-Rad).

### 2.7 Cell proliferation assays

Cell proliferation of aorta *in vivo* was detected by proliferating cell nuclear antigen (PCNA) using Western blot (PCNA antibody. Sigma, St. Louis, MO, USA). Cell proliferation of cultured VSMCs *in vitro* was assessed by incubating with WST-1 reagent for 1 hour which colorimetric analyze the number of viable cells by the cleavage of tetrazolium salts (Cell Proliferation Reagent WST-1, Roche), and the absorbance at 450 nm was measured in ELISA plate reader (Bio-Rad 680).

### 2.8 Cell migration assays

Scratch wound assay was employed for cell migration assessment as previously described [30]. Briefly, VSMCs were cultured in 6-well plate. Once 95% confluent, cross lines, i.e. three horizontal and one vertical strips, were scratched with a sterile 200 μl pipette tip. Cells were washed twice with PBS and then cultured in DMEM supplemented with 10% FBS and 5% CO_2_ at 37°C for 24 or 48 hours. Images were photographed by microscope (Olympus IX-71) and the distance of cell migration was analyzed by using Image Pro Plus 6.0 software.

### 2.9 Statistical analysis

Each experiment was performed at least four times, and all values are expressed as the mean ± *SD*. The *student’s t*-test was used to compare two groups. One-way ANOVA and the subsequent post-hoc, Student-Newman-Keuls (S-N-K) test, were used to compare 3 or more groups. A value of *P* < 0.05 was regarded as statistically significant.

## Results

### 3.1 In vivo, the mean blood pressure, NPY concentration, opening angle, and media thickness of hypertension in pregnant model

**Fig 1:**
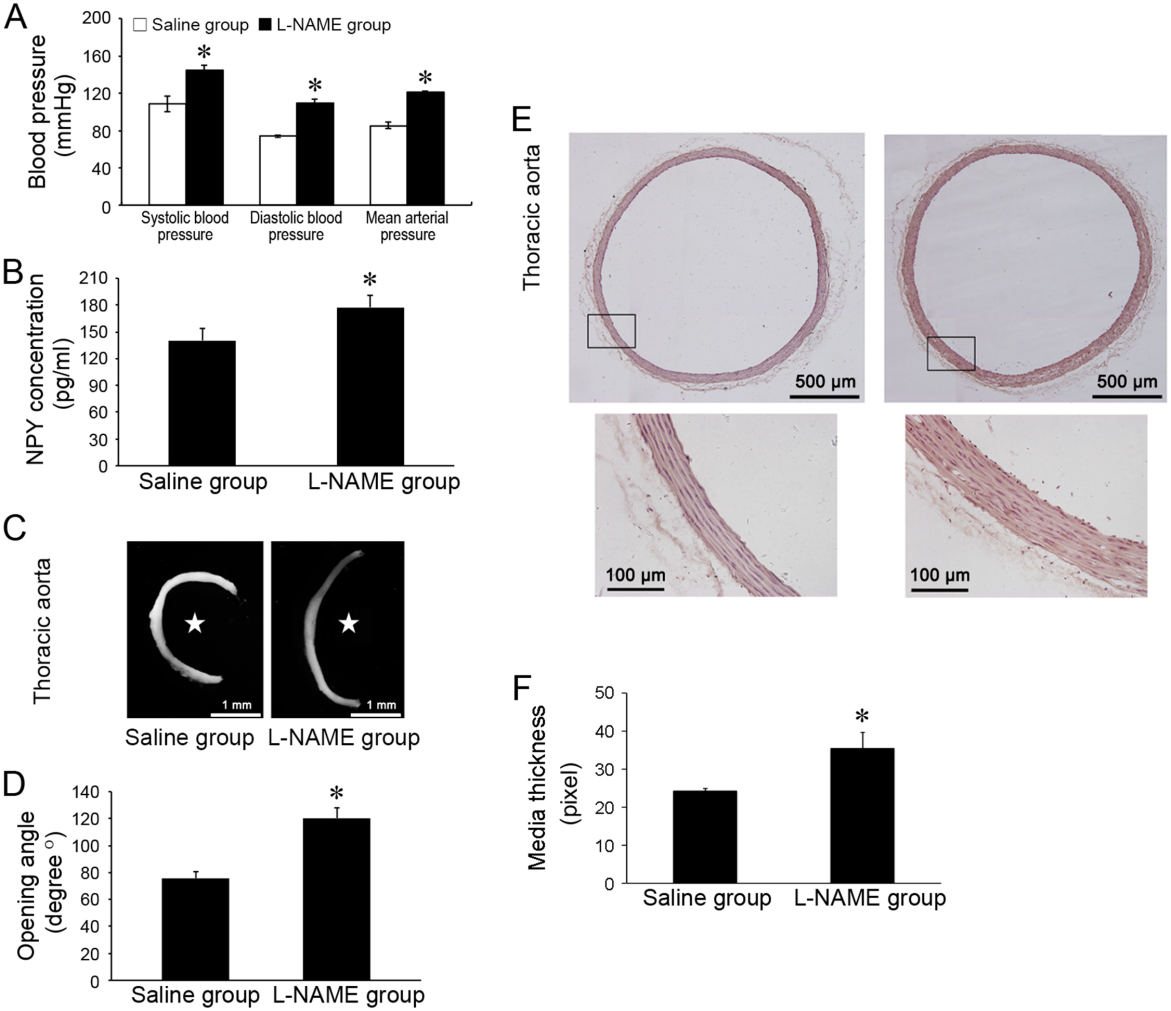
The changes of blood pressure, plasma NPY concentration, and opening angle of thoracic aorta in the gestational hypertensive rat model. (A) The systolic blood pressure, diastolic blood pressure and mean arterial pressure were significantly increased in L-NAME group compared with saline control group (*n* = 4). (B) The plasma concentrations of NPY in L-NAME group was higher than saline control group (*n* = 4). (C) Representative microscopic images illustrate opening angles. Star-marked the inner wall of arteries. (D) The opening angle of aorta in L-NAME group was significantly increased compared with that in saline control group (*n* = 4, and for each n there were 4 rings). (E) Representative microscopic images of H&E staining illustrate media thickness. (F) The thickness of aortic media in L-NAME group was significantly increased compared with that in saline control group (saline control group, *n* = 4. L-NAME group, *n* = 5, and for each *n* there were 4 microscopic images). Shown values are the mean ± *SD*, * *P*<0.05 *v*.*s*. the saline control.

To establish model of hypertension in pregnancy, rats were injected with L-NAME with control groups injected with saline. On P20.5, the systolic blood pressure, diastolic blood pressure and mean arterial pressure of L-NAME group were all significantly higher than that of the control group (Fig 1A). Plasma concentration of NPY in L-NAME group was 177.5778 ± 12.915 pg/ml, and increased 26.44% comparing to the saline control group (140.44675 ± 13.1187 pg/ml) (Fig 1B). The opening angle of L-NAME group was 120.4039 ± 7.765°, and was larger than the control group (75.76833 ± 5.119°) (Fig 1C and 1D). Consistently, the arterial media of L-NAME group was significantly thicker than that of the control group (Fig 1E and 1F).

These data (S1 Table) demonstrate that NPY concentration increases in L-NAME induced hypertension in pregnant model. The increased open angle in responsive to hypertension in pregnancy suggestes the non-uniform remodeling and anisotropic growth of the arterial wall [27, 28].

### 3.2 In vivo, the expressions of Y receptor, c-Fos, PCNA, STAT3, and phosphorylations of STAT3 at Ser727 and Tyr705 in the thoracic aorta of hypertension in pregnant model

**Fig 2:**
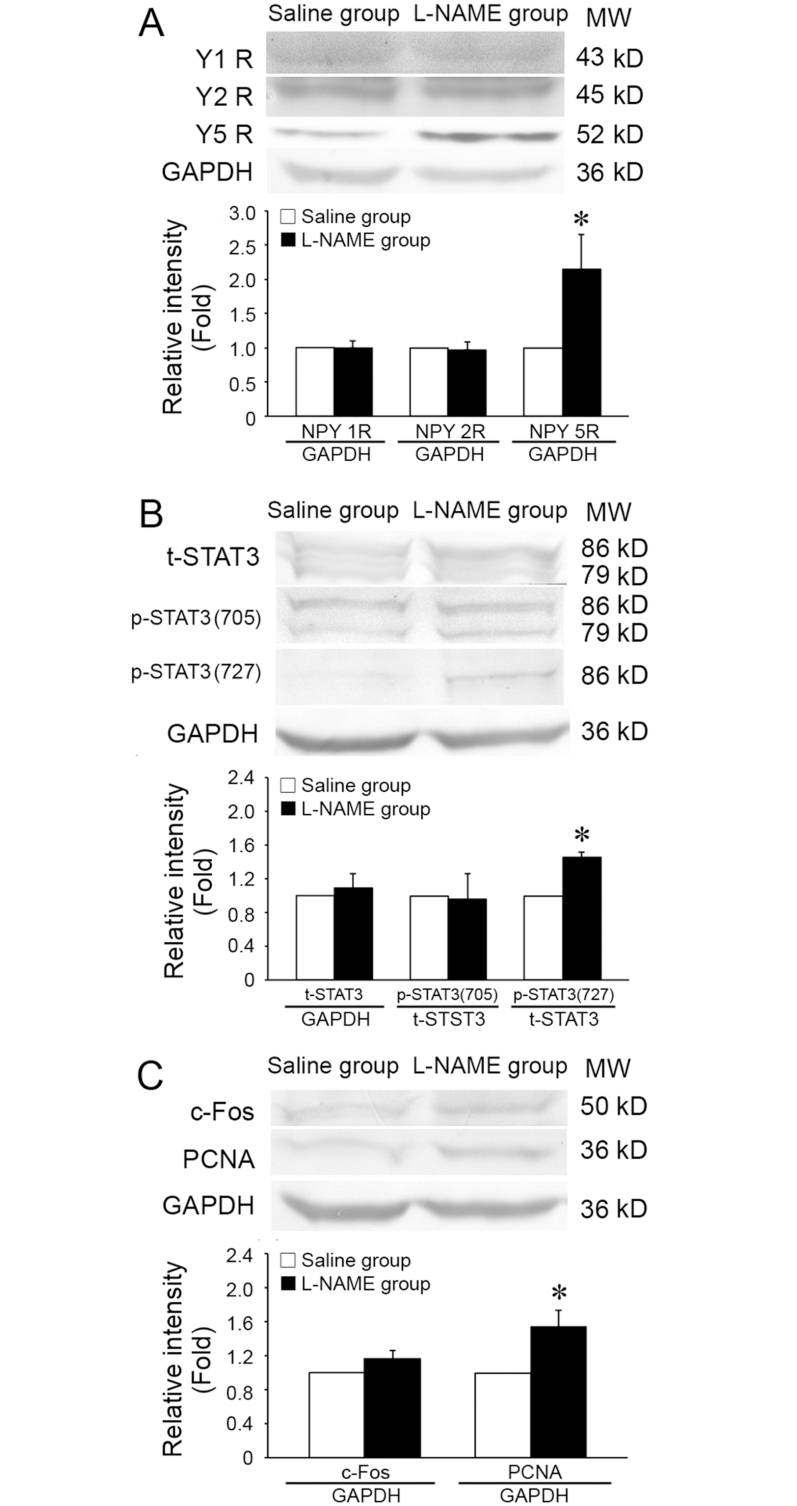
The expressions of NPY receptors, phosphorylation of STAT3, and expressions of c-Fos and PCNA in thoracic aorta of gestational hypertensive rats. (A) The expression of NPY5R was significantly increased, but not NPY1R and NPY2R in L-NAME group compared with that in saline control group (NPY1R and NPY2R, *n* = 4. NPY5R, *n* = 3). (B) The phosphorylation of STAT3 at Ser727 residue was significantly increased in L-NAME group, while the expression of total STAT3 and phosphorylation of STAT3 at Tyr705 were not changed (STAT3 at Tyr705, *n* = 4. total STAT3 and STAT3 at Ser727, *n* = 3). (C) Compared with saline control group, the expressions of c-Fos and PCNA were increased in L-NAME group (c-Fos, *n* = 3. PCNA, *n* = 4). Shown values are the mean ± *SD*, * *P*<0.05 *v*.*s*. the saline control.

There were no significant difference on the expressions of Y1R, Y2R, total STAT3, and phosphorylation of STAT3 at Tyr705 between the L-NAME group and the saline control group (Fig 2A and 2B). The expression of Y5R was significantly increased in the L-NAME group in comparison with that in the saline group (2.14- fold) (Fig 2A). The phosphorylation of STAT3 at Ser727 as well as the expression of c-Fos were also increased to 1.46-fold and 1.16- fold, respectively (Fig 2B and 2C). The expression of PCNA which reflects the proliferation of cells was 1.54-fold higher in the L-NAME group than that in the saline group (Fig 2C).

These results (S2 Table) in L-NAME model indicates the increased periferal concentration of NPY and prolifearion of vascular cells. Then, the role and molecular mechanisms of NPY in VSMC proliferation were further detected in vitro.

### 3.3 In vitro, VSMC proliferation modulated by NPY treatment (S3 Table)

**Fig 3:**
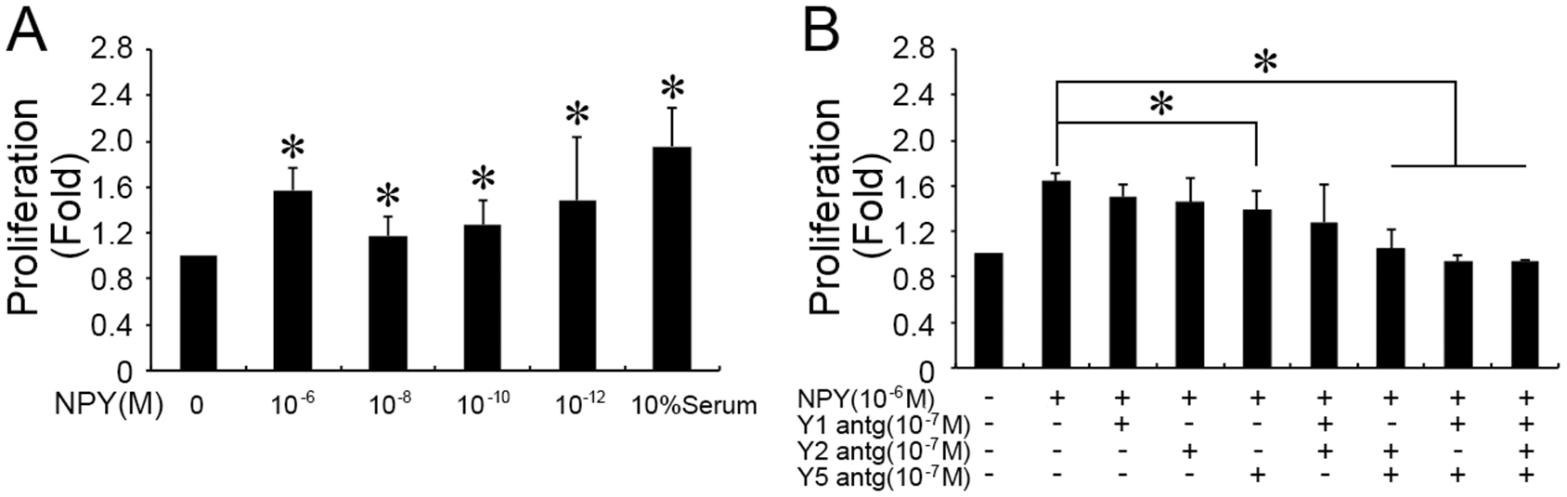
NPY stimulated the proliferation of cultured VSMCs, and the NPY receptors were involved in this process. (A) VSMCs were stimulated by NPY at different concentration for 24 hours. The last column showed 10% serum data (*n* = 4). (B) VSMCs were pre-incubated with single or combinational NPY receptor antagonists (NPY 1, 2 and 5 receptor antagonists), and then treated with 10^−6^ mol/L NPY (*n* = 4). Shown values are the mean ± *SD*, * *P*<0.05 *v*.*s*. the respective control.

After incubating with NPY (10^−6^, 10^−8^, 10^−10^, 10^−12^ mol/L) for 24 hours, VSMC proliferation was examined by WST (Fig 3A). The first peak value (1.57 fold over the control) was shown when VSMCs were treated with NPY at the concentration of 10^−6^ mol/L (*P* < 0.05), while 10^−12^ mol/L NPY resulted in a 1.48 fold increase (*P* < 0.05 compared with no NPY control).

To evaluate the receptors of NPY on VSMC proliferation, VSMCs were pre-incubated with Y1, Y2 and Y5 receptor antagonists, respectively, and then treated with 10^−6^ mol/L NPY. Fig 3B showed that single or combinational pre-incubation of Y1R and Y2R antagonists had no specific effect on NPY induced VSMC proliferation. While, single pre-incubation of Y5R antagonist remarkbly reduced VSMC proliferaion induced by the NPY. Moreover, the combinational pre-incubation of Y5R antagonist with Y1R and/or Y2R antagonists can completely eliminate the NPY’s effect (Fig 3B).

### 3.4 In vitro, VSMC migration modulated by NPY treatment (S4 Table)

**Fig 4:**
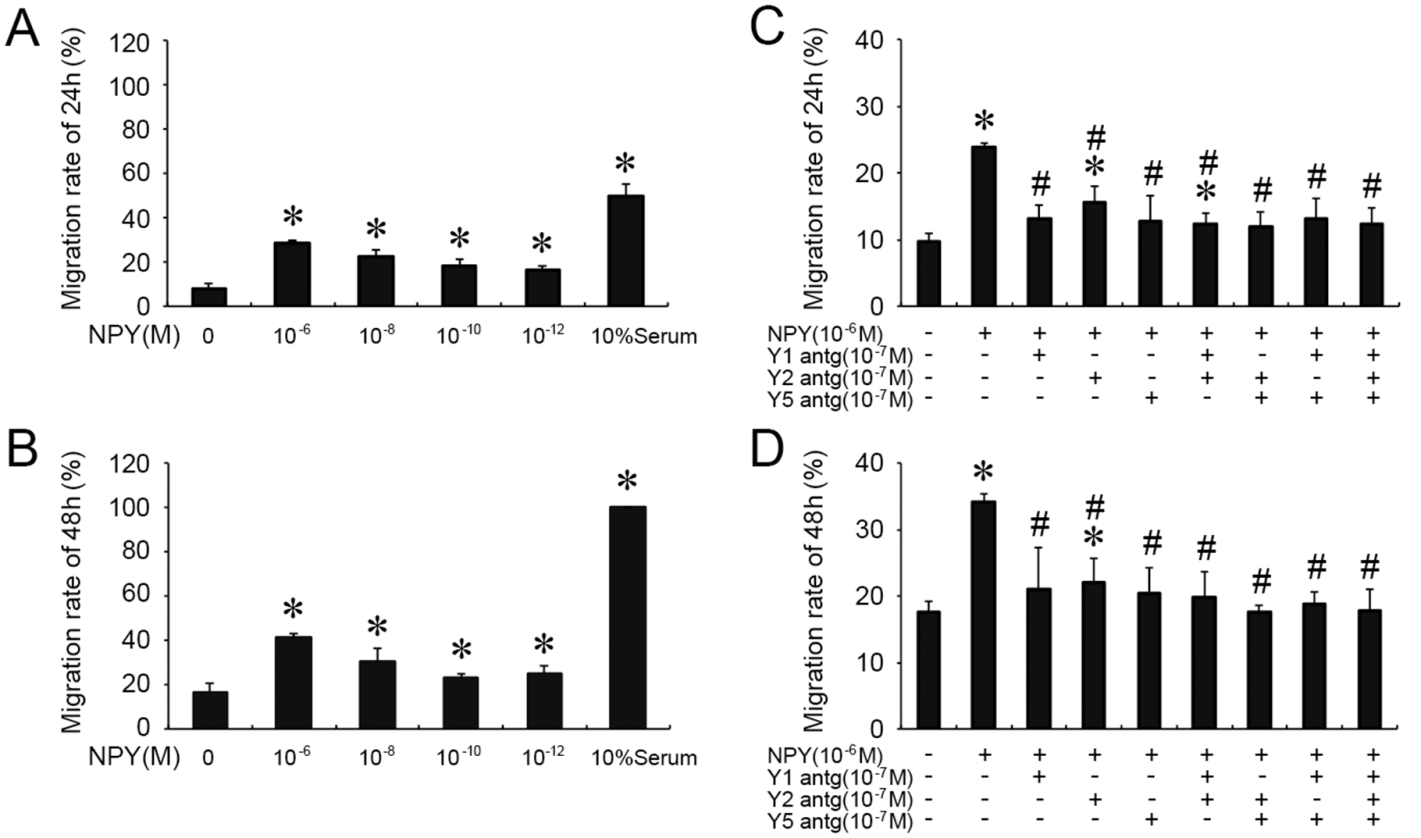
NPYs induced the migration of cultured VSMCs, and the NPY receptors were involved in this process. (A), (B) NPYs at different concentration treated for 24 hours and 48 hours induced the migration of cultured VSMCs (*n* = 4). The migration rate of VSMCs is defined as the ratio of migrating distance, after 24 hours’ treatment, to the initial distance scratched by sterile 200 μl pipette tip. Shown values are the mean ± *SD*, * *P* < 0.05 *v*.*s*. the respective control; (C), (D) VSMCs were pre-incubated with single or combinational NPY receptor antagonists (NPY 1, 2 and 5 receptor antagonists), and then treated with 10^−6^ mol/L NPY for 24 hours and 48 hours (*n* = 4). Shown values are the mean ± *SD*, * *P* < 0.05 *v*.*s*. respective the first column (0 mol/L NPY); ^#^ *P* < 0.05 f *v*.*s*. respective the second column (10^−6^ mol/L NPY).

The effects of NPY on the VSMC migration, another important cellular function, were also evaluated. After incubation with NPY (10^−6^, 10^−8^, 10^−10^ and 10^−12^ mol/L) for 24 hours, the migration rate of VSMCs, defined as the ratio of migrating distance to the initial distance scratched by sterile 200 μl pipette tip, was measured. Fig 4A showed that compared with no NPY control, NPY at all four concentrations significantly increased the migration rate of VSMCs (28.711 ± 0.7%, 22.44 ± 3.19%, 18.34 ± 2.76%, and 16.35 ± 1.66%, respectively). NPY at 10^−6^ mol/L increased the VSMC migration most remarkable in this assay for 48 hours (41.03 ± 2.24%) (Fig 4B). NPY at 10^−8^ M also significantly increased the VSMC migration for 48 hours (30.09 ± 6.36%, *P* < 0.05).

To evaluate the receptors involved in NPY-induced migration, VSMCs were pre-incubated with Y1, Y2 and Y5 receptor antagonists, respectively, and then treated with 10^−6^ mol/L NPY. The results showed that the single or combinational pre-inculation of antagonist all significantly blocked the effects of NPY on VSMC migration (Fig 4C and 4D).

### 3.5 In vitro, expressions of Y5 receptor, c-Fos, PCNA and phosphorylation of STAT3 modulated by NPY treatment

**Fig 5:**
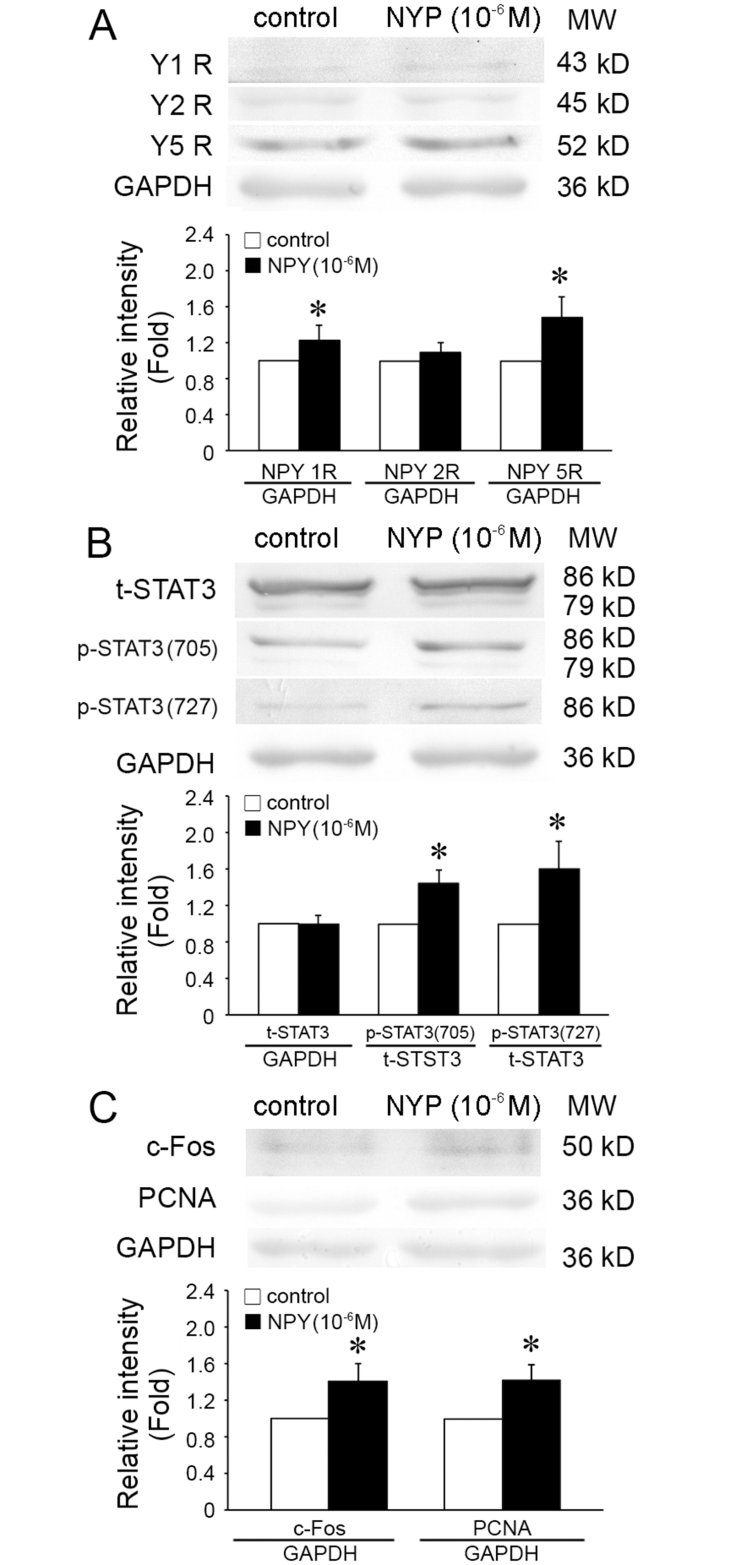
NPY modulated the expressions of NPY1R, NPY5R, phosphorylation of STAT3, and expression of c-Fos in cultured VSMCs. (A) After treatment of NPY for 24 hours, the expressions of NPY1R and NPY5R, but not NPY2R, were significantly increased in VSMCs (NPY2R, *n* = 4. NPY1R and NPY5R, *n* = 5). (B) After treatment of NPY for 24 hours, the phosphorylations of STAT3 at Tyr705 and Ser727 were both increased (*n* = 6). (C) After treatment of NPY for 24 hours, the expressions of c-Fos and PCNA were significantly increased (*n* = 4). Shown values are the mean ± *SD*, * *P*<0.05 *v*.*s*. the respective control.

Fig 5 showed that the expressions of Y1R and Y5R, and c-Fos were significantly increased by NPY treatment compared with control group. Phosphorylations of STAT3 at Ser727 and Tyr705, were also induced by NPY treatment. The expressions of Y1R and Y5R were up-regulated by 123% and 147.4%, respectively. STAT3 phosphorylation at Ser727 and Tyr705 increased 44.17% and 60%, respectively. The expression of c-Fos also increased 1.4025 fold upon NPY stimulation. PCNA in VSMCs treated with 10^−6^ mol/L NPY also increased 41.75% (*P* < 0.05) in comparison with no NPY control (Fig 5C).

These results (S5 Table) suggest that NPY effect on these molecular signals and induce VSMCs proliferation in vitro is consistent with that in vivo.

## Discussion

NPY of the periphery is released from the adrenal medulla and the sympathetic nerves [2, 31]. In human subjects and animals, plasma NPY levels elevate in response to various stressful conditions, such as cold exposure, tissue injury, particularly accompanied hypoxia [32], and to sympathetic hyperactivities, such as hypertension and congestive heart and renal failure [33]. In the present study, we demonstrated that plasma NPY levels were remarkably increased in gestational hypertensive model. Accompanied with the increased NPY concentration, the opening angle of thoracic aorta was also elevated, which indicates the pathophysiological remodeling of the arterial wall [26, 27].

During hypertension, the abnormally increased proliferation, differentiation and migration of VSMCs induce the non-uniform remodeling and anisotropic growth of the arterial wall, which causes the increased opening angle [26, 34, 35]. Using in vitro cultured VSMCs, we demonstrated the potential role of in vivo increased NPY on VSMC functions, and revealed that NPY stimulates VSMC proliferation and migration ranging from femtomolar to nanomolar concentrations.

In vitro, NPY induces EC migration, proliferation and capillary tube formation, and multiple Y receptors are involved in modulating different cell functions [8]. Y receptor (Y1, Y2, Y5) antagonists affected proliferation (Y1+Y2, Y1+Y5, Y2+Y5) and capillary tube formation on Matrigel (Y1 + Y2 + Y5) of ECs [8]. The stress-induced increases in circulating NPY can lead to vasoconstriction of resistance vessels [36, 37], and hypertensive responses [35], which can be blocked by Y1R antagonists. Furthermore, both NPY release and its Y1R mediated vasoconstriction are greater in males than in females because of testosterone-mediated upregulation of NPY gene expression [6, 38]. Our results also demonstrated that NPY promotes the migration and proliferation of female rat VSMCs. Indeed, not only migration but also proliferation were most effectively modulated by 10^−6^ mol/L NPY, with both Y1 and Y5 receptors being up-regulated. Consistently, the Y1, Y2 and Y5 receptor antagonists were applied to confirm the specific receptor subtypes required for NPY’s response. Interesting, single Y5R antagonist can reduce the VSMC proliferation, with more significantly effect after combined with other NPY receptor antagonists. It implies that Y5R plays the main role in modulating VSMC proliferation.

STAT3 is a transcription factor, which plays crucial roles in modulating VSMC proliferation, migration and apoptosis [39, 40]. Our results revealed that phosphorylations at different residues of STAT3 may participate in NPY-induced proliferation and migration of VSMCs. Phosphorylation of STAT3 on Tyr705 residue plays important roles in its nuclear translocation and transcriptional activating function [41]. STAT3 phosphorylation at Ser727 residue has been shown to increase the transcriptional potential of STAT3, and the cooperation of both tyrosine (705) as well as serine (727) phosphorylation is necessary for full activation of Stat3 [41, 42]. Shimada et al. [21] has showed that NPY increased phosphorylation STAT3 at Ser 727 residue in stromal vascular cells from brown adipose tissue. Transcriptional activation of eukaryotic genes often requires the cooperative action of many proteins [43]. C-Fos encodes a 62 kDa protein, which is overexpressed in a variety of cancers and plays an important role in many cellular functions. Overexpression of c-Fos and c-Jun strongly strengthened STAT3-driven IRE transactivation as well as transactivation of the human intercellular adhesion molecule (ICAM)-1 promoter [44]. We demonstrated that NPY modulates VSMC proliferation and migration via up-regulating the expression of c-Fos and the activation of STAT3. The mechanisms of c-Fos expression and STAT3 phosphorylation on NPY-induced VSMC functions during gestational hypertensive need to be analyzed in the future research.

The molecular pathogenesis of hypertension in pregnancy is very complex and still far from fully clarified. Here, our results suggested that NPY participated in the vascular remodeling during hypertension in pregnancy, but there are still many questions need further researches. For examples, L-NAME animal model is dependent on the block of nitric oxide synthase [23, 24], which cannot reflect the complete spectrum of pathophysiological changes associated with hypertension in pregnancy; the mechanism undergoes the differences between the in vivo and in vitro experiments, whether some other peptides are also involved in the vascular remodeling induced by hypertension in pregnancy; ECs exist in the intima of vessel wall which are directly exposed to the circulating NPY, whether NPY also participates in EC dysfunction during hypertension in pregnancy. The proteomic analyses are planed to use in the future to detect the protein profiles of plasma and artery in model hypertension in pregnancy, which help us deeply understand the molecular mechanisms of vascular remodeling in response to hypertension in pregnancy.

In summary, our results revealed that the elevated plasma concentration of NPY during hypertension in pregnancy may induce VSMC proliferation mainly via Y5 receptor, which subsequently modulate STAT3 and c-Fos signaling pathways, and then induces vascular remodeling. These results also suggest that NPY mainly acts on VSMCs in vitro via Y1, Y5 receptors and in vascular tissues in vivo via Y5 receptor.

## Supporting Information

S1 Table Minimal data of Fig 1. Table 1-A The changes of blood pressure in the gestational hypertensive rat model. Table 1-B The changes of plasma NPY concentration in the gestational hypertensive rat model. Table 1-C＆D The changes of opening angle of thoracic aorta in the gestational hypertensive rat model. Table 1-E＆F The changes of aortic media thickness in the gestational hypertensive rat model.
(PDF)

S2 Table Minimal data of Fig 2. Table 2-A The expression of NPY1R , NPY2R and NPY5R in thoracic aorta. Table 2-B The expression of STAT3, p-STAT3 in thoracic aorta. Table 2-C The expression of c-Fos, PCNA in thoracic aorta.
(PDF)

S3 Table Minimal data of Fig 3. Table 3-A The proliferation of cultured VSMCs were stimulated by NPY for 24 hours. Table 3-B The proliferation of cultured VSMCs were stimulated by pre-incubated with NPY receptor antagonists.
(PDF)

S4 Table Minimal data of Fig 4. Table 4-A The migration of cultured VSMCs were stimulated by NPY for 24 hours. Table 4-B The migration of cultured VSMCs were stimulated by NPY for 48 hours. Table 4-C The migration of cultured VSMCs were stimulated by NPY receptor antagonists for 24 hours. Table 4-D The migration of cultured VSMCs were stimulated by NPY receptor antagonists for 48 hours.
(PDF)

S5 Table Minimal data of Fig 5. Table 5-A The expression of NPY1R , NPY2R and NPY5R in cultured VSMCs. Table 5-B The expression of STAT3, p-STAT3 in cultured VSMCs. Table 5 C The expression of c-Fos, PCNA in cultured VSMCs.
(PDF)
