# Supplementary material for: Neuropeptide Y Stimulates Proliferation and Migration of Vascular Smooth Muscle Cells from Pregnancy Hypertensive Rats via Y1 and Y5 Receptors
Source: PLoS One. 2015 Jul 1;10(7):e0131124. doi: 10.1371/journal.pone.0131124 (PMC4488588; doi:10.1371/journal.pone.0131124)
Supplement: S1 Table — Table 1-A The changes of blood pressure in the gestational hypertensive rat model. Table 1-B The changes of plasma NPY concentration in the gestational hypertensive rat model. Table 1-C＆D The changes of opening angle of thoracic aorta in the gestational hypertensive rat model. Table 1-E＆F The changes of aortic media thickness in the gestational hypertensive rat model. (PDF) [file pone.0131124.s001.pdf]

The changes of blood pressure

mmHg	Systolic blood pressure		Diastolic blood pressure		Mean arterial pressure	
	Saline group	L-NAME group	Saline group	L-NAME group	Saline group	L-NAME group
N1	101	145	80	103	87	117
	137	141	64	116	89	124
	116	150	82	107	93	121
	118	145.33	75.33	108.67	89.67	120.67
N2	91	136	77	120	82	125
	105	144	72	105	83	118
	97	137	73	107	81	117
	97.67	139	74	110.67	82	120
N3	109	148	71	106	84	120
	110	152	80	101	90	118
	114	153	68	108	83	123
	111	151	73	105	85.67	120.33
N4	105	147	78	114	87	125
	109	136	64	113	79	121
	109	142	75	114	86	123
	107.67	141.67	72.33	113.67	84	123
**mean**	**108.58**	**144.25**	**73.67**	**109.5**	**85.33**	**121**
SD	**8.46**	**5.2**	**1.31**	**3.64**	**3.25**	**1.36**
***P* =**		**0.31718196**		**0.61741671**		**0.87111009**

The changes of NPY concentrations

(From Beijing DORUN International Technology Co., Ltd)

pg/ml	Saline group	L-NAME	Saline/L-NAME
N1	148.782	188.334	1.26583861
N2	145.525	177.696	1.221068545
N3	146.606	184.899	1.261196677
N4	120.874	159.382	1.318579678
**mean**	**140.44675**	**177.57775**	**1.2669483**
SD	**13.1186117**	**12.91506764**	**0.049009348**
***P*=**		**0.000201343**	

The changes of opening angles

degree °	Saline group	L-NAME
N1	75.298325	127.3272
N2	70.6268	111.772
N3	82.84118	115.9203
N4	74.379015	126.5961
**mean**	**75.78633**	**120.4039**
SD	**5.118930316**	**7.765045402**
***P*=**		**0.002380487**

The changes of aortic media thickness

Pixel	Saline group	L-NAME
N1	23.64368365	33.82834395
N2	24.93062633	42.76504777
N3	23.94824841	34.72993631
N4	24.70334395	33.28646497
N5		32.52998938
**mean**	**24.30647558**	**35.42795648**
SD	**0.609559542**	**4.179207467**
***P*=**		**0.000622062**
