# Supplementary material for: Neuropeptide Y Stimulates Proliferation and Migration of Vascular Smooth Muscle Cells from Pregnancy Hypertensive Rats via Y1 and Y5 Receptors
Source: PLoS One. 2015 Jul 1;10(7):e0131124. doi: 10.1371/journal.pone.0131124 (PMC4488588; doi:10.1371/journal.pone.0131124)
Supplement: S2 Table — Table 2-A The expression of NPY1R , NPY2R and NPY5R in thoracic aorta. Table 2-B The expression of STAT3, p-STAT3 in thoracic aorta. Table 2-C The expression of c-Fos, PCNA in thoracic aorta. (PDF) [file pone.0131124.s002.pdf]

The expression of NPY1R , NPY2R and NPY5R in thoracic aorta

	NPY1R/GAPDH		NPY2R/GAPDH		NPY5R/GAPDH	
	Saline group	L-NAME group	Saline group	L-NAME group	Saline group	L-NAME group
N1	1	0.853772	1	0.805551	1	1.563011
N2	1	1.030239	1	1.028853	1	2.456183
N3	1	0.959791	1	0.931073	1	2.41064
N4	1	1.114224	1	1.083891	1	
**mean**	**1**	**0.9895065**	**1**	**0.962342**	**1**	**2.143278**
SD	**0**	**0.11033352**	**0**	**0.12214614**	**0**	**0.503041633**
***P*=**		**0.427706839**		**0.28007369**		**0.008504987**

The expression of STAT3, p-STAT3 in thoracic aorta

	T-STAT3/GAPDH		p-STAT3 705/T-STAT3		p-STAT3 727/T-STAT3	
	Saline group	L-NAME group	Saline group	L-NAME group	Saline group	L-NAME group
N1	1	1.263217	1	0.723999435	1	1.387695
N2	1	0.869826	1	0.592494977	1	1.504995
N3	1	1.147434	1	1.046801233	1	1.487212
N4			1	1.468412328		
**mean**	**1**	**1.093492333**	**1**	**0.95792699**	**1**	**1.459967333**
SD	**0**	**0.165068465**	**0**	**0.39019507**	**0**	**0.051617351**
***P*=**		**0.234007612**		**0.41820186**		**0.00011411**

The expression of c-Fos, PCNA in thoracic aorta

	c-Fos/GAPDH		PCNA/GAPDH	
	Saline group	L-NAME group	Saline group	L-NAME group
N1	1	1.296638	1	1.773716
N2	1	1.12516	1	1.386186
N3	1	1.06416	1	1.304726
N4			1	1.687869
**mean**	**1**	**1.161986**	**1**	**1.53812425**
SD	**0**	**0.098416194**	**0**	**0.19715916**
***P*=**		**0.040227549**		**0.00323305**
