# Supplementary material for: Neuropeptide Y Stimulates Proliferation and Migration of Vascular Smooth Muscle Cells from Pregnancy Hypertensive Rats via Y1 and Y5 Receptors
Source: PLoS One. 2015 Jul 1;10(7):e0131124. doi: 10.1371/journal.pone.0131124 (PMC4488588; doi:10.1371/journal.pone.0131124)
Supplement: S3 Table — Table 3-A The proliferation of cultured VSMCs were stimulated by NPY for 24 hours. Table 3-B The proliferation of cultured VSMCs were stimulated by pre-incubated with NPY receptor antagonists. (PDF) [file pone.0131124.s003.pdf]

The proliferation of cultured VSMCs were stimulated by NPY for 24 hours

	0 M NPY	10^{-6} M NPY	10^{-8}M NPY	10^{-10}M NPY	10^{-12}M NPY	10% serum
N1	1	1.63	1.080373	1.502415459	2.394958	2.23
N2	1	1.816425121	1.4251208	1.215730337	1.095328	2.22705314
N3	1	1.4614	1.0314961	1.080373832	1.188764	1.8
N4	1	1.379775281	1.1483146		1.647343	1.569
N5					1.0682415	
**mean**	**1**	**1.5719001**	**1.171326**	**1.26617321**	**1.478927**	**1.956513285**
SD	**0**	**0.193468604**	**0.175847**	**0.21549512**	**0.562913**	**0.32794804**
***P*=**		**0.000521037**	**0.049627**	**0.02541437**	**0.046807**	**0.000292808**

The proliferation of cultured VSMCs were stimulated by pre-incubated with NPY receptor antagonists

		NPY (10^{-6} M)	NPY receptor antagonists (10^{-7} M) + NPY(10^{-6} M)						
	0 M NPY	DMSO	Y1R antagonist	2R antagonist	5R antagonist	1+2R antagonists	2+5R antagonists	1+5R antagonists	1+2+5R antagonists
N1	1	1.626140	1.534954	1.632219	1.306991	1.097264	0.881459	0.860182	0.951368
N2	1	1.606232	1.371105	1.130312	1.257790	0.974504	0.915014	0.997167	0.923513
N3	1	1.742382	1.614958	1.506925	1.634349	1.739612	1.257618	0.908587	0.941828
N4	1	1.560137	1.494845	1.536082	1.357388	1.305842	1.120275	0.941581	0.934708
**mean**	**1**	**1.633723**	**1.503966**	**1.451385**	**1.389130**	**1.279306**	**1.043591**	**0.926879**	**0.937854**
SD	**0**	**0.077535**	**0.101677**	**0.220641**	**0.168461**	**0.335971**	**0.177489**	**0.057560**	**0.011747**
***P*=**			**0.088717**	**0.169934**	**0.038650**	**0.085555**	**0.000889**	**0.000006**	**0.000002**
