# Supplementary material for: Neuropeptide Y Stimulates Proliferation and Migration of Vascular Smooth Muscle Cells from Pregnancy Hypertensive Rats via Y1 and Y5 Receptors
Source: PLoS One. 2015 Jul 1;10(7):e0131124. doi: 10.1371/journal.pone.0131124 (PMC4488588; doi:10.1371/journal.pone.0131124)
Supplement: S4 Table — Table 4-A The migration of cultured VSMCs were stimulated by NPY for 24 hours. Table 4-B The migration of cultured VSMCs were stimulated by NPY for 48 hours. Table 4-C The migration of cultured VSMCs were stimulated by NPY receptor antagonists for 24 hours. Table 4-D The migration of cultured VSMCs were stimulated by NPY receptor antagonists for 48 hours. (PDF) [file pone.0131124.s004.pdf]

The migration of cultured VSMCs were stimulated by NPY for 24 hours

	0 M NPY	10-6 M NPY	10-8M NPY	10-10M NPY	10-12M NPY	10% serum
N1	3.21%	29.18%	22.16%	22.57%	16.84%	56.08%
N2	9.35%	28.93%		17.44%	14.97%	49.29%
N3	8.07%	29.23%	18.68%	18.45%	18.86%	51.62%
N4	10.16%	27.51%	26.48%	14.89%	14.72%	41.63%
**mean**	**7.70%**	**28.71%**	**22.44%**	**18.34%**	**16.35%**	**49.66%**
SD	**2.70%**	**0.70%**	**3.19%**	**2.76%**	**1.66%**	**5.24%**
***P*=**		**0.000012**	**0.002520**	**0.003081**	**0.003228**	**0.000017**

The migration of cultured VSMCs were stimulated by NPY for 48 hours

	0 M NPY	10^{-6} M NPY	10^{-8}M NPY	10^{-10}M NPY	10^{-12}M NPY	10% serum
N1	11.95%	43.79%	28.62%	23.23%	25.29%	100.00%
N2	14.62%	38.55%		25.07%	21.89%	100.00%
N3	18.95%	41.68%	24.59%	21.19%	29.45%	100.00%
N4	20.94%	40.10%	37.05%		22.08%	100.00%
**mean**	**16.61%**	**41.03%**	**30.09%**	**23.17%**	**24.68%**	**100.00%**
SD	**4.08%**	**2.24%**	**6.36%**	**1.94%**	**3.55%**	**0.00%**
***P*=**		**0.000044**	**0.018265**	**0.026415**	**0.024523**	**0.00000001**

The migration of cultured VSMCs were stimulated by NPY receptor antagonists for 24 hours

		NPY (10^{-6} M)	NPY receptor antagonists (10^{-7} M) + NPY(10^{-6} M)						
	0 M NPY	DMSO	1R antagonist	2R antagonist	5R antagonist	1+2R antagonist	2+5R antagonist	1+5R antagonist	1+2+5R antagonist
N1	10.28%	24.04%	11.22%	15.38%	11.86%	13.51%	9.61%	14.28%	12.02%
N2	10.97%	23.60%	12.64%	19.12%	7.42%	13.89%	13.91%	11.14%	15.52%
N3	8.47%	23.29%	13.35%	13.80%	16.09%	11.26%	13.85%	16.72%	11.70%
N4	8.68%	24.55%	15.68%	14.00%	15.20%	10.66%	10.61%	10.28%	9.71%
**mean**	**9.60%**	**23.87%**	**13.22%**	**15.57%**	**12.64%**	**12.33%**	**12.00%**	**13.11%**	**12.23%**
SD	**1.22%**	**0.55%**	**1.86%**	**2.47%**	**3.93%**	**1.61%**	**2.21%**	**2.96%**	**2.42%**
***P*= (*v.s.*10^{-6} M** NPY)			**0.001064**	**0.008330**	**0.010234**	**0.001088**	**0.003027**	**0.007673**	**0.003561**
***P*= (*v.s.* 0 M NPY)**		**0.000255**	**0.082949**	**0.004860**	**0.320036**	**0.035343**	**0.151260**	**0.141755**	**0.099545**

The migration of cultured VSMCs were stimulated by NPY receptor antagonists for 48 hours

		NPY (10^{-6} M)	NPY receptor antagonists (10^{-7} M) + NPY(10^{-6} M)						
	0 M NPY	DMSO	1R antagonist	2R antagonist	5R antagonist	1+2R antagonist	2+5R antagonist	1+5R antagonist	1+2+5R antagonist
N1	19.38%	34.90%	28.38%	23.71%	20.97%	25.49%	18.79%	20.90%	21.65%
N2	17.18%	33.79%	18.61%	19.76%	15.65%	18.55%	17.75%	19.62%	16.94%
N3	18.18%	32.24%	23.33%	26.38%	25.19%	17.50%	16.76%	18.09%	18.79%
N4	15.73%	35.24%	13.43%	18.23%	19.76%	17.86%	17.10%	16.64%	13.68%
**mean**	**17.62%**	**34.04%**	**20.93%**	**22.02%**	**20.39%**	**19.85%**	**17.60%**	**18.81%**	**17.76%**
SD	**1.55%**	**1.35%**	**6.40%**	**3.71%**	**3.93%**	**3.78%**	**0.89%**	**1.85%**	**3.34%**
***P*= (*v.s.*10^{-6} M NPY)**			**0.031509**	**0.014839**	**0.010352**	**0.003556**	**0.000098**	**0.000868**	**0.003576**
***P*= (*v.s.* 0 M NPY)**		**0.000752**	**0.265371**	**0.045801**	**0.223446**	**0.214834**	**0.977337**	**0.110507**	**0.880669**
