# Supplementary material for: Neuropeptide Y Stimulates Proliferation and Migration of Vascular Smooth Muscle Cells from Pregnancy Hypertensive Rats via Y1 and Y5 Receptors
Source: PLoS One. 2015 Jul 1;10(7):e0131124. doi: 10.1371/journal.pone.0131124 (PMC4488588; doi:10.1371/journal.pone.0131124)
Supplement: S5 Table — Table 5-A The expression of NPY1R , NPY2R and NPY5R in cultured VSMCs. Table 5-B The expression of STAT3, p-STAT3 in cultured VSMCs. Table 5 C The expression of c-Fos, PCNA in cultured VSMCs. (PDF) [file pone.0131124.s005.pdf]

The expression of NPY1R , NPY2R and NPY5R in cultured VSMCs

	NPY1R/GAPDH		NPY2R/GAPDH		NPY5R/GAPDH	
	0 M NPY	10^{-6} M NPY	0 M NPY	10^{-6} M NPY	0 M NPY	10^{-6} M NPY
N1	1	1.47	1	1.1	1	1.26
N2	1	1.24	1	1.25	1	1.73
N3	1	1.11	1	1	1	1.66
N4	1	1.29	1	1.01	1	1.22
N5	1	1.04			1	1.5
**mean**	1	1.23	1	1.09	1	1.474
SD	0	0.16718253	0	0.11575837	0	0.229738982
***P*=**		0.01520306		0.17095303		0.001724811

The expression of STAT3, p-STAT3 in cultured VSMCs

	T-STAT3/GAPDH		p-STAT3 705/T-STAT3		p-STAT3 727/T-STAT3	
	0 M NPY	10^{-6} M NPY	0 M NPY	10^{-6} M NPY	0 M NPY	10^{-6} M NPY
N1	1	0.819	1	1.34	1	1.25
N2	1	0.97	1	1.66	1	1.55
N3	1	1.03	1	1.34	1	1.75
N4	1	1.02	1	1.57	1	1.52
N5	1	0.993	1	1.47	1	1.41
N6	1	1.13	1	1.27	1	2.12
**mean**	**1**	**0.993666667**	**1**	**1.441666667**	**1**	**1.6**
SD	**0**	**0.101655628**	**0**	**0.151712447**	**0**	**0.303446865**
***P*=**		**0.881742883**		**3.17588E-05**		**0.000678106**

The expression of c-Fos, PCNA in cultured VSMCs

	c-Fos/GAPDH		PCNA/GAPDH	
	0 M NPY	10^{-6} M NPY	0 M NPY	10^{-6} M NPY
N1	1	1.28	1	1.36
N2	1	1.44	1	1.29
N3	1	1.66	1	1.35
N4	1	1.23	1	1.67
**mean**	**1**	**1.4025**	**1**	**1.4175**
SD	**0**	**0.19362765**	**0**	**0.171148084**
***P*=**		**0.005961233**		**0.002769544**
